# Highly reliable behaviors

**DOI:** 10.1186/s13584-015-0048-1

**Published:** 2015-09-29

**Authors:** Erin S. DuPree

**Affiliations:** Joint Commission Center for Transforming Healthcare, 1 Renaissance Blvd, Oakbrook Terrace, IL 60181 USA

**Keywords:** Safety culture, Nurse-physician conflicts, Disruptive behavior, High reliability

## Abstract

A lack of respect between nursing and medical disciplines can lead to a lack of trust and disruptive behaviors that are a significant part of the culture of health care today. In order to ensure the best care for all patients, a systematic approach to defining desired and undesired behaviors is a place to begin. A systems view requires an appreciation of local culture and operations. Understanding the underlying root causes in different departments and specialties allows for the development and implementation of sustainable solutions which will ultimately change and transform an organization. Leadership action and commitment at the highest strategic level is essential for this to occur.

## Background

Calming, soothing, settling, disciplined and well-behaved. Are these the feelings and descriptors that come to mind with every nurse-physician communication? In the best of circumstances these words, antonyms to “disruptive,” [1] describe interactions between health care professionals. Unfortunately, nurses and physicians do not always communicate in this fashion, for a myriad of reasons that certainly includes production pressures and rapid changes in the industry today. Additionally, misunderstanding of roles and a lack of respect can also contribute to ineffective and, at times, harmful communication and behavioral patterns.

In a recent IJHPR article, authors Berman-Kishony, *et al.* [2] make a significant contribution to the understanding of behaviors in health care delivery. They survey residents and nurses to identify similarities and differences in behaviors and the effectiveness of conflict management tools. This allows for an analysis of the various micro cultures that exist in different areas of an organization. They find that the behaviors map to three “buckets”: hidden, expressed, and sophisticated. Importantly, their analysis shows that there are many antecedents for these behaviors: many root causes. It follows that the interventions and solutions will need to vary yet most behavioral interventions have historically involved a one size fits all approach, with policies and training. While certainly important in establishing norms and expectations, policies and training rarely solve problems or change culture in a sustainable fashion. After a code of conduct, what can be done to change the culture?

## The Joint Commission approach

The Joint Commission noted the importance of behaviors among professionals years ago with the establishment of leadership standards [3] and a Sentinel Event Alert [4] that specify how behaviors such as reluctance or refusal to answer questions and not answering calls can negatively impact patient care and the importance of having a code of conduct in place. The standards point leaders to the fundamental items that organizations should strive to have in place. Yet, the AHRQ Patient Safety Culture survey and the Institute for Safe Medication Practices Workplace Intimidation survey show limited, at best mixed, improvement over the past decade [5, 6]. The everyday culture in health care continues to be rampant with incivility [7], even with policies in place. Given the prevalence of these behaviors in health care, lack of substantial progress, and growing body of evidence that links poor behaviors with poor patient outcomes, leaders can work to determine the root causes that continue to produce unacceptable behaviors in order to change the system.

Improving business processes and patient outcomes requires a systematic approach. Effective process improvement includes both a leadership commitment and the engagement of front line staff that know the operations of a unit the best. Likewise, improving culture in an organization requires a systematic approach with a leadership commitment to model behavior, every time, in every situation. Leadership commitment includes letting staff know that they can stand up to unacceptable behaviors exhibited by those around them and that the staff will have the explicit support of leadership. Leadership commitment starts with the alignment of a vision across all disciplines from the governing body, to the chief executive officer and all physician and nursing leaders. What is desirable behavior? What is unacceptable behavior? A vision defines the best possible culture. There are the clear examples of unacceptable behavior, such as destroying organizational property, but then there are the examples that are not always straightforward and sometimes vary depending on the recipient. What about superficial listening, rude comments, public criticism or a condescending tone? As Felblinger noted, individuals do not always come to an organization with a clear idea of what constitutes considerate conduct in general, as inconsiderate conduct is tolerated in many health care organizational cultures, so incivility reigns throughout much of health care [7]. In addition, there are regional and national norms of behavior. A brusque, get-it-done tone might be acceptable to many health care workers in New York City, yet would be seen as rude and offensive in other areas of the country and to workers that have moved to New York City from elsewhere. The vast majority of leadership’s attention, and the research to date, is focused in a reactive manner on the 1–2 % of physicians and staff that behave like no one else, the “bad apples” [8]. Yet focusing on these few individuals ignores the context and many factors that give rise to these behaviors, thus enabling the severe disruptions. It does not allow for the development of effective solutions to address the culture issues in healthcare. A narrow definition of disruptive behavior that only includes the obvious issues such as yelling and screaming ignores the daily, unit-specific interactions between nurses and physicians that define the local, unit culture, within a specific regional and national context.

Physicians are often rewarded, inadvertently, for aggressive, goal-oriented behavior that is not civil. They are able to continue to act on behalf of individual patients without regard for the system and organization in which they work. Typically these “unsafe conditions” are not defined, measured, or analyzed in an organization. A focus on the most extreme behavioral situations and people leads to a recurring movie with the same issues, just different players.

On the contrary, a systematic approach to defining, measuring and analyzing behaviors, both desired and undesired, can lead to effective, targeted solutions. Targeted solutions target root causes. Root causes vary from unit to unit, and organization to organization, as the personalities, work flows, and regional norms differ. Highly reliable patient care requires an understanding that operations vary throughout an organization and require a sensitivity to operations [9]. This is consistent with what we have learned about improving clinical outcomes - the solutions vary, depending on the contributing factors. We know that for hand hygiene, for instance, there are over 20 main causes of failure to wash hands [10]. Each cause requires a different, targeted solution. Different units and different organizations have different causes. This applies not only to patient care processes, but also to the behaviors that are inherent in the execution of patient care.

A systems view acknowledges that disruptive behaviors are more frequent than outright physical and verbal abuse. The current aim of eliminating the most egregious behaviors leads to a reactive approach to the problem. Disruptive behaviors will persist with this mindset. The elimination of disruptive behaviors, broadly defined, is essential for changing the culture of health care organizations. Until there is zero tolerance for all disruptive behaviors along with a vision of consistent considerate, thoughtful behavior, there will be limited improvement.

A systems level problem requires a systematic approach, such as Six Sigma DMAIC (Define-Measure-Analyze-Improve-Control), with leadership commitment and the involvement of frontline staff. Disruptive behavior is not simple to define. To accurately define (Define) the daily incivility requires an understanding of the many viewpoints that exist (e.g. across different disciplines, genders, nationalities, and races). It requires health care leaders to take a look in the mirror, which is not customary in health care today. In contrast, businesses outside of health care have had processes in place for many years, with most Fortune 500 companies using a 360° review as part of performance management since the 1990s. Organizations can move to development of an accurate measurement system and analysis (Measure, Analyze) once leaders and staff have established aims and operational definitions of both desired and inappropriate behavior. What are the contributing factors in different departments?

## Conclusions

Berman-Kishony’s work takes an important step in understanding different departments and specialties. This analysis is important for the development and implementation of interventions (Improve) that will eliminate disruptive behaviors. A systems view of behaviors and culture will lead to solutions that *prevent* disruptive behaviors, instead of spending valuable time *reacting* to bad behavior. Targeted solutions allow for ongoing monitoring of the behaviors critical to excellent patient care and ultimately, sustainability (Control). Over time, operational definitions may need to be adjusted, as a unit and an organization learns from failures; the “Define” phase is re-entered over time. This approach supports organizational learning, which is at the heart of a reliability culture.

In order to develop interventions and solutions for a culture of safety in health care, leaders can take on “culture” as a strategic priority, starting with definitions of desired and undesired behaviors that take various backgrounds and perspectives into account so that measurement is accurate, allowing for robust analysis and understanding of the root causes. These definitions evolve from a discovery process that involves engagement of leaders and staff across an organization. What are the model behaviors? What is offensive? Why are those behaviors offensive and why do they occur? What are the conditions that lead to those behaviors? Leadership commitment and alignment at all levels enables an organization to learn what the root causes of both desired and undesired behaviors are in their setting. With this understanding the development and implementation of targeted solutions is possible, leading to a culture that will enable health care to become highly reliable, delivering excellent care to every patient, every time.
